# Association between Anterior Surgical Approach and Dysphagia Severity in Patients with Cervical Spinal Cord Injury

**DOI:** 10.3390/jcm12093227

**Published:** 2023-04-30

**Authors:** Min Cheol Chang, Dae Yeong Kim, Jin-Woo Choi, Ho Yong Choi, Jin-Sung Park, Donghwi Park

**Affiliations:** 1 Department of Rehabilitation Medicine, Yeungnam University Hospital, Daegu 42415, Republic of Korea; 2 Department of Physical Medicine and Rehabilitation, Ulsan University Hospital, University of Ulsan College of Medicine, Ulsan 44033, Republic of Korea0736043@uuh.ulsan.kr (J.-W.C.); 3 Department of Neurosurgery, Kyung Hee University Hospital at Gangdong, College of Medicine, Kyung Hee University, Seoul 05278, Republic of Korea; 4 Department of Neurology, School of Medicine, Kyungpook National University, Kyungpook National University Chilgok Hospital, Daegu 41944, Republic of Korea; 5 Department of Rehabilitation Medicine, Daegu Fatima Hospital, Ayangro 99, Dong gu, Daegu 41199, Republic of Korea

**Keywords:** deglutition, spinal cord injury, dysphagia, tracheostomy

## Abstract

Introduction: Early detection and management of dysphagia are essential for preventing aspiration pneumonia and reducing mortality in patients with cervical spinal cord injury (C-SCI). In this study, we identified risk factors for dysphagia in patients with C-SCI by analyzing the correlation between the clinical factors and the severity of dysphagia, not the presence or absence of dysphagia. Combined with the analysis results of previous studies, we thought that this additional analysis method could more accurately reveal the risk factors for dysphagia in patients with C-SCI. Methods: The presence and severity of dysphagia in patients with C-SCI was evaluated using a modified videofluoroscopic dysphagia scale (mVDS) and penetration–aspiration scale (PAS). All included patients with C-SCI performed a video fluoroscopic swallowing study (VFSS). Clinical factors such as age, sex, the presence of tracheostomy, spinal cord independence measure (SCIM), pulmonary function test (PFT), including forced vital capacity (FVC), forced expiratory volume in 1 s (FEV1), FVC/FEV1, maximal inspiratory pressure (MIP), and maximal expiratory pressure (MEP), American Spinal Cord Injury Association (ASIA) score, Berg Balance Scale (BBS), and operation method were investigated. Results: In the multivariate regression analysis, the anterior surgical approach was the only clinical factor that had a significant correlation in both mVDS and PAS, which represents the severity of dysphagia in C-SCI patients (*p* < 0.05). Conclusion: The anterior surgical approach was correlated with the severity of dysphagia in patients with C-SCI. Considering this, as one of the risk factors affecting dysphagia in patients with C-SCI, surgical method may also need to be considered. Additionally, we recommend that clinicians should pay particular attention to the potential for development of dysphagia in patients who received anterior cervical surgery. However, further prospective studies with larger sample sizes are needed for more accurate generalization.

## 1. Introduction

Patients with cervical spinal cord injury (C-SCI) usually suffer from partial or complete motor weakness in the upper and lower extremities and impairment of sensory and autonomic functions [1]. Dysphagia is relatively one of the common complications in patients with C-SCI [2]. Previous studies have reported that the prevalence of dysphagia in patients with C-SCI varies from 16–80% [2,3,4]. Dysphagia in patients with C-SCI can increase morbidity and mortality by increasing the risk of aspiration pneumonia [5,6]. In particular, aspiration pneumonia is one of the major causes of morbidity and mortality in patients with C-SCI [7]. Therefore, early detection and management of dysphagia are essential to prevent aspiration pneumonia and reduce mortality in patients with C-SCI.

In several previous studies, old age, the presence of tracheostomy, vocal quality, the anterior surgical approach for the cervical spine (lower level of anterior cervical spine operation), higher level of neurological injury (American Spinal Cord Injury Association (ASIA) grade A or B), and reduced performance on pulmonary function testing (such as low forced expiratory volume for 1 s (FEV1) and low peak cough flow (PCF)) have been reported as risk factors for dysphagia in patients with C-SCI [2,7,8,9,10,11,12,13].

However, most previous studies divided patients with C-SCI into two groups according to the presence or absence of dysphagia and identified several risk factors for dysphagia in patients with C-SCI [2,3,4,7]. According to the presence or absence of dysphagia, patients with C-SCI were classified into dysphagia and non-dysphagia groups based on the presence of penetration (penetration–aspiration scale (PAS) score ≥ 2; contrast enters the airway, remains above the vocal folds, and is ejected from the airway) [2,14]. However, considering that even healthy asymptomatic individuals can have a PAS score ≥ 2, this uniform binary classification may lead to bias in the derivation of the results [15]. In this study, instead of using the binary classification according to the presence or absence of dysphagia, we identified risk factors for dysphagia in patients with C-SCI by analyzing the correlation between the clinical factors and the severity of dysphagia as a continuous variable. Combined with the analysis results of previous studies, we thought that this additional analysis method could more accurately reveal the risk factors for dysphagia in patients with C-SCI.

## 2. Materials and Methods

### 2.1. Study Design and Population

This study was approved by the Institutional Review Board (IRB) of Ulsan University Hospital (IRB number: 22-04-037). Data on patients with C-SCI at Ulsan University Hospital between January 2016 and August 2022 were collected retrospectively. We obtained clinical data such as age, sex, the cause of C-SCI, the presence of tracheostomy, spinal cord independence measure (SCIM) scores, completeness of SCI, presence of Levin tube, neurological level, pulmonary function test (PFT) results, including forced vital capacity (FVC), FEV1, the ratio of FEV1 to FVC (FVC/FEV1), maximal inspiratory pressure (MIP), and maximal expiratory pressure (MEP), American Spinal Cord Injury Association (ASIA) scores, Berg Balance Scale (BBS) scores, videofluoroscopic swallowing study (VFSS) data, and surgical approach (anterior or posterior).

The inclusion criteria were: (1) C-SCI, which is defined by certified board radiologic specialists as confirming a high signal intensity at the cervical spinal cord using T2-weighted C-SCI magnetic resonance imaging (MRI), and it is consistent with cervical myelopathy; (2) weakness or sensory disturbance in both upper and lower extremities due to C-SCI, and (3) VFSS for dysphagia evaluation.

The exclusion criteria were: (1) age under 19 years of age; (2) no swallowing test or PFT; (3) dysphagia due to neurologic conditions diagnosed by neurologists, including stroke, traumatic brain injury, anoxic brain injury, brain tumor, motor neuron disease, Parkinson’s disease, or Alzheimer’s disease; (4) other surgical or medical histories that could cause dysphagia, such as laryngeal or tongue cancer or vocal cord paralysis; (5) other surgical or medical histories that were not related to the C-SCI; (6) dysphagia before C-SCI; (7) an interval of >14 days between the VFSS, PFT, and clinical evaluations (such as SCIM and ASIA). We investigated consecutive patients with C-SCI who met the inclusion and exclusion criteria. This study was performed in accordance with the Declaration of Helsinki for human experiments, and the requirement for informed consent was waived because of the study’s retrospective nature.

### 2.2. Oropharyngeal Dysphagia Evaluation

VFSS was performed using a fluoroscopy device (Luminos dRF MAX; Siemens, Munchen, Germany) and recorded in a video file to evaluate the presence and severity of dysphagia. During VFSS, the patient swallowed the following substances in order: water, nectar (51–350 cP), rice porridge (351–1750 cP), and boiled rice (>1750 cP) [16,17]. Each patient received an initial 3 mL bolus, followed by two 5 mL boluses. Fluids (thick, nectar-like, and thin) were delivered using 10 mL syringes; patients with dysphagia of grades I, II, or III were fed with spoons. All substances were mixed with barium, and then the patient swallowed the substances in a sitting position. Dynamic fluoroscopy images were recorded at a rate of 30 frames per second and were obtained from the lateral views. Results (the evaluation of oropharyngeal dysphagia) were analyzed using a modified videofluoroscopic dysphagia scale (VDS) and PAS; aspiration was considered present when the PAS score was >5 [18]. The modified VDS is a scale of 100 points (minimum–maximum, 0–100), scored by a physiatrist based on several parameters in the VFSS video. The higher the modified VDS score, the more severe the dysphagia. Modified VDS has demonstrated its good inter-rater reliability (Cronbach α value, 0.886) in previous studies [19,20,21,22]. The PAS is a scale of 8 points (minimum–maximum, 1–8), scored by a physiatrist based on the degree of aspiration in the VFSS video. The higher the PAS score, the more severe the aspiration of food into the airways. PAS has demonstrated its good inter-reliability (0.890–0.910) and intra-rater reliability (0.900–0.970) in previous studies [23].

### 2.3. Spinal Cord Independence Measure Scores

The SCIM is a scale used to measure the achievement of daily function in patients with SCI [24]. The third version of SCIM (SCIM III) contains 19 tasks organized into three subscales: self-care, respiration and sphincter management, and mobility [24]. We investigated and compared all three subscale scores as well as the total score. The SCIM was evaluated through interviews and observation by the patients’ occupational therapists [24].

### 2.4. Pulmonary Function Test

Spirometry was conducted according to American Thoracic Society guidelines Vmax 22 (Sensor-Medics, Yorba Linda, CA, USA). The following parameters were evaluated: FVC, FEV1, and FEV1/FVC [25]. All spirometric values are expressed as percentages of the predicted values. In addition, maximal inspiratory pressure (MIP) and maximal expiratory pressure (MEP) were also measured using spirometry (Pony FX, COSMED Inc., Rome, Italy) with a rigid tube-type mouthpiece [26].

### 2.5. American Spinal Cord Injury Association Score

The ASIA International Standards for Neurological Classification of Spinal Cord Injury form was used to evaluate spinal cord injuries (Atlanta, GA, Revised 2011, Updated 2015. Published with permission of the ASIA, Richmond, VA, USA) [27]. The sensory examination evaluated 28 specific dermatomes bilaterally for pinprick (generally a clean safety pin) and light touch (generally a piece of cotton) sensations. Each examination component was recorded for each dermatome and laterality [28]. A grade of 0 denoted a lack of sensation, 1 denoted an impaired or altered sensation, and 2 denoted a normal sensation. Furthermore, a normal unilateral sensory examination consists of 28 dermatomes, each with 2 points for light touch and 2 points for pinprick, yielding a total of 112 points [28]. A total score of 224 bilaterally was considered a fully normal sensory examination [28]. The motor examination graded five specific muscle groups in the upper extremities and five in the lower extremities, representing major cervical and lumbar myotomes [28]. Motor strength was graded using a universal six-point scale (0–5). Motor strength was recorded bilaterally in each muscle group [28]. The maximum bilateral motor score in a healthy individual is 100, with 50 for scoring 5/5 in all right upper and lower extremity myotomes and another 50 for the left [28].

### 2.6. The Berg Balance Scale

The BBS is a 14-item scale that quantitatively assesses balance and risk of falls in older community-dwelling adults through direct observation of their performance [29]. The items were scored from 0–4, with a score of 0 representing an inability to complete the task and a score of 4 representing independent item completion [29]. The global score was calculated from 56 possible points. The BBS scores of the patients with C-SCI were measured by a physical therapist.

### 2.7. Surgical Approach

The surgical methods were investigated in patients with C-SCI who underwent cervical spine surgery. One of our authors (H.Y.C.), a spine specialist neurosurgeon, classified the patients’ surgical methods by investigating the surgical record. The surgical method was divided into an anterior approach and a posterior approach according to the location of the skin incision and the approach to the cervical spine. Basically, the type of surgical approach was determined by the characteristics of injury as well as surgeons’ preferences. In case of anterior pathology such as ruptured intervertebral disc or burst fracture combined with significant bony retropulsion, anterior approach was used. On the other hand, a mainly posterior lesion such as fractured posterior bony fragment compressing spinal cord or locked facet joints that were irreducible by traction or anterior approach, a posterior approach was used. In cases necessitating decompression for multi-level (more than three level), a posterior approach was also used. In case of highly unstable injuries, surgery with combined anterior-posterior approach was performed. Cases of combined anterior-posterior approach surgeries were excluded from this study.

### 2.8. Statistical Analysis

For evaluation of the correlation between the severity of dysphagia and various clinical factors, *p*-values were calculated using Pearson’s simple correlation test. If the *p*-value was less than 0.1, it was then used as an independent variable in multivariate regression analysis. Using clinical factors that showed a *p*-value less than 0.1 with the severity of dysphagia (PAS or mVDS), multivariate regression analysis using stepwise methods was performed to evaluate the correlation with the severity of dysphagia in patients with C-SCI. Statistical significance was set at a *p*-value less than 0.05 (typically ≤ 0.05). Statistical analyses were performed using MedCalc and SPSS software (version 22.0; IBM Corp., Armonk, NY, USA).

## 3. Results

### 3.1. Patient Characteristics

We investigated a total of 230 patients, and among them, 174 patients who did not meet the inclusion criteria were excluded. Finally, a total of 56 patients with C-SCI were enrolled in this study. All patients with C-SCI enrolled in this study were admitted to our hospital. Among them, 26 were male, and 13 were female (mean age, 64.36 ± 12.43 years). Fifteen patients underwent cervical spine surgery with an anterior approach, and 41 underwent cervical spine surgery with a posterior approach. Regarding VFSS, the mean mVDS was 32.51 ± 20.29, and the mean PAS was 4.03 ± 2.81. Among the 56 patients, 8 had complete SCI and 48 had incomplete SCI. The other demographic data of the patients with C-SCI are shown in Table 1. All dysphagia-related parameters (VFSS and the presence of Levin tube), respiratory-related factors (PFT, MIP, MEP, and the presence of tracheostomy), and severity of C-SCI (SCIM scores, ASIA scores, and BBS scores) were all measured within 2 weeks of all measurements.

### 3.2. The Correlation between the mVDS and Clinical Factors

In Pearson’s simple correlations test, the *p*-values of the surgical approach, BBS, and ASIA motor score were less than 0.1 (Table 2). Other factors, such as sex, were not correlated with mVDS. In the multivariate regression analysis using these five parameters to identify any association with mVDS in patients with C-SCI, the surgical approach was the only significant clinical factor associated with mVDS in patients with C-SCI (*p* < 0.05; Table 3).

### 3.3. The Correlation between the PAS and Clinical Factors

In Pearson’s simple correlations test, the *p*-values of the surgical approach, the presence of tracheostomy, and the presence of Levin tube were less than 0.1 (Table 2). Other factors, such as sex, were not correlated with mVDS. In the multivariate regression analysis using these three parameters to identify any association with PAS in patients with C-SCI, the surgical approach was the only significant clinical factor associated with PAS in patients with C-SCI (*p* < 0.05; Table 4).

## 4. Discussion

This study’s result revealed that both PAS and mVDS, indicating the severity of dysphagia, showed a statistically significant correlation only with the anterior surgical approach. Patients with C-SCI who underwent the anterior surgical approach had significantly more severe dysphagia than those who underwent the posterior surgical approach. However, the presence of tracheostomy, pulmonary function test (FVC or FEV1), and age, which were significantly correlated with the presence of dysphagia in patients with C-SCI in previous studies, did not have significant correlations with the severity of dysphagia in this study [2,14]. To the best of our knowledge, this is the first study to evaluate the risk factors for dysphagia in patients with C-SCI by analyzing the correlation between the clinical factors and the severity of dysphagia. As mentioned before, combined with the analysis results of previous studies, we thought that this additional analysis method could more accurately reveal the risk factors for dysphagia in patients with C-SCI.

While the prevalence of dysphagia may be potentially under-reported, previous studies have reported a dysphagia rate of approximately 79% within the first week after the anterior surgical approach for the cervical spine [30,31]. Dysphagia may be caused by prevertebral soft tissue swelling, hematoma, bleeding, nerve injuries such as recurrent laryngeal nerve, or inflammation associated with anterior cervical hardware irritation or esophageal retraction [32,33].

Furthermore, several clinical trials have been performed to prevent dysphagia after anterior cervical spine surgery. First, steroids have been applied intraoperatively to reduce dysphagia risk during ACDF [34,35,36,37]. While a few previous studies showed a decreased rate of dysphagia after applying steroids during anterior cervical spine surgery [36,37], prospective randomized controlled trials (RCTs) have not shown any benefit of intravenous or local intra-operative administration of steroids for swallowing function [31,34,35]. However, Jeyamohan et al. [37] found that intra-operative administration of dexamethasone significantly improved the function of swallowing and edema of the airway but did not affect long-term swallowing status. Moreover, retropharyngeally local administration of steroids to prevent postoperative dysphagia was associated with a delayed fusion or decreased radiographic fusion rate after anterior cervical spine surgery [37]. Second, intra-operative administration of local anesthetics to the retropharyngeal space did not improve swallowing difficulty after anterior cervical spine surgery [31].

Despite the disadvantages of the anterior approach, including an increased incidence of dysphagia, compared with posterior surgical approaches, the anterior surgical approach can directly decompress the structures most commonly responsible for cervical myelopathy and relieve compression of the cervical spinal cord caused by cervical kyphosis [38]. Moreover, the anterior approach is associated with less postoperative neck pain and can provide superior restoration of cervical sagittal alignment through anterior column support and prevent further degeneration and neurological deterioration over the fused segment [38,39].

In actual clinical practice, the correlation between the severity of dysphagia and anterior cervical spine surgery tends to be under-reported in patients with C-SCI [40]. In fact, when performing surgery on patients with C-SCI using an anterior approach, there may be a high possibility of affecting the anatomical structures (e.g., laryngeal muscles and nerves) associated with the swallowing process. Moreover, this study’s results have shown that the anterior surgical approach was the only risk factor significantly correlated with the severity of dysphagia in C-SCI patients. Therefore, additional studies such as surgical methods or specific medication administration are necessary to reduce complications such as dysphagia after surgery in patients with C-SCI.

This study has a few limitations. First, due to the characteristics of a retrospective study in a single tertiary university hospital, unlike the RCT study, many patients with C-SCI could not be enrolled, and variables affecting the severity of dysphagia could not be completely controlled. Moreover, since the cohort of C-SCI patients who operated with an anterior approach in this study was somewhat smaller than the cohort of C-SCI with a posterior approach, it cannot be considered that the interpretation of the results was completely free of potential bias. In addition, the duration of the interval between surgery and VFSS was not consistent due to the retrospective study’s nature. However, as mentioned before, most previous studies that investigated the risk factors for dysphagia in C-SCI patients were also retrospective studies. Moreover, to compensate for the shortcomings of previous studies using binary classification, we investigated the risk factors for dysphagia in patients with C-SCI by analyzing the correlation between clinical factors and the severity of dysphagia, not the presence of dysphagia. Using these methods combined with the analysis results of previous studies, we thought we could more accurately reveal the risk factors for dysphagia in patients with C-SCI. However, further prospective studies with larger sample sizes are needed in the future for more accurate generalization.

## 5. Conclusions

The anterior surgical approach was correlated with the severity of dysphagia (both mVDS and PAS) in patients with C-SCI in this study. Considering this, as one of the risk factors affecting dysphagia in patients with C-SCI, a surgical method may also need to be considered. Additionally, we recommend that clinicians should pay particular attention to the potential for development of dysphagia when patients receive anterior cervical surgery. However, since the cohort of C-SCI patients who operated with an anterior approach in this study was somewhat smaller than the cohort of C-SCI with a posterior approach, it cannot be considered that the interpretation of the results was completely free of potential bias. Therefore, for more accurate generalization, further prospective studies with larger sample sizes are needed.

## Figures and Tables

**Table 1:** Characteristics of cervical spinal cord injury patients who were included in this study.

Characteristics	Patients with C-SCI
Epidemiology	
Age	69.00 (min–max: 34–85)
Sex (Male:Female)	37:19
The severity of SCI	
SCIM	
SCIM_self care score	3.00 (min–max: 0–18)
SCIM_respiration score	23.00 (min–max: 0–40)
SCIM_mobility score	2.00 (min–max: 0–37)
SCIM_total score	26.00 (min–max: 2–90)
ASIA	
ASIA_motor score	58.00 (min–max: 0–99)
ASIA_sensory score	64.00 (min–max: 0–112)
ASIA impairment scale A (complete): B, C, and D (incomplete)	8:48
Berg balance scale (BBS)	2.50 (min–max: 0–56)
Respiratory-related factors	
Pulmonary function test	
FVC (% predicted)	61.00 (min–max: 14–99)
FEV1 (% predicted)	74.00 (min–max: 20–112)
FEV1/FVC ratio	82.00 (min–max:44–100)
Maximal inspiratory pressure (%)	78.50 (min–max: 0–166)
Maximal expiratory pressure (%)	68.50 (min–max: 0–124)
Tracheostomy (Yes: No)	44:12
Surgical approaches	
Operation approach (Anterior:Posterior)	15:41
Dysphagia severity	
mVDS	29.00 (min–max: 3–76)
PAS	4.00 (min–max: 1–8)
Interval between surgery and VFSS (days)	14.00 (min–max: 5–358)
Levin Tube (Yes: No)	44:12

Median (minimum–maximum). SCIM: spinal cord independence measure; ASIA: American Spinal Cord Injury Association; FVC: forced vital capacity; FEV1: forced expiratory volume for 1 s; mVDS: modified videofluroscopic dysphagia scale; C-SCI: cervical spinal cord injury; PAS: Penetration-aspiration scale.

**Table 2:** Correlations between the severity of dysphagia and other clinical factors.

	mVDS	PAS
Surgical approach	−0.544 (*p* = 0.001)	−0.658 (*p* < 0.001)
BBS	0.395 (*p* = 0.056)	0.005 (*p* = 0.984)
ASIA_motor score	−0.356 (*p* = 0.068)	−0.283 (*p* = 0.190)
ASIA_sensory score	−0.243 (*p* = 0.222)	−0.163 (*p* = 0.458)
Completeness of SCI	−0.134 (*p* = 0.505)	−0.199 (*p* = 0.363)
Tracheostomy	0.276 (*p* = 0.114)	0.596(*p* = 0.001)
Levin Tube	0.286 (*p* = 0.101)	0.532 (*p* = 0.003)
FVC (%)	−0.235 (*p* = 0.238)	−0.223 (*p* = 0.306)
FEV1 (%)	−0.210 (*p* = 0.293)	−0.263 (*p* = 0.226)
FEV1/FVC	−0.213 (*p* = 0.286)	−0.195 (*p* = 0.372)
MIP (%)	−0.201 (*p* = 0.509)	−0.404 (*p* = 0.247)
MEP (%)	−0.448 (*p* = 0.125)	−0.520 (*p* = 0.123)

BBS: berg balance scale; ASIA: American Spinal Cord Injury Association; FVC: forced vital capacity; FEV1: forced expiratory volume for 1 s; mVDS: modified videofluroscopic dysphagia scale; SCI: spinal cord injury; MIP: maximal inspiratory pressure; MEP: maximal expiratory pressure; PAS: penetration-aspiration scale.

**Table 3:** Multivariate regression analysis with the Stepwise method of the severity of dysphagia (modified Videofluoroscopic dysphagia scale (mVDS)) and clinical factors in the patients with cervical spinal cord injury.

	B	Standard Error	*p*-Value	Exp (B)	95% CI	
					Lower Bound	Upper Bound
Surgical approach	−40.656	12.301	0.004 *	−0.637	−66.734	−14.579

* *p*-value < 0.05.

**Table 4:** Multivariate regression analysis with the Stepwise method of the severity of dysphagia (Penetration-aspiration scale (PAS)) and clinical factors in the patients with cervical spinal cord injury.

	B	Standard Error	*p*-Value	Exp (B)	95% CI	
					Lower Bound	Upper Bound
Surgical approach	−3.763	0.851	<0.001 *	−0.648	−5.510	−2.017

* *p*-value < 0.05.

## Data Availability

The data that support the findings of this study are available from the corresponding author upon reasonable request.

## References

[1] Wolf C., Meiners T.H. (2003). Dysphagia in patients with acute cervical spinal cord injury. Spinal Cord.

[2] Lee S.J., Huh S., Ko S.H., Min J.H., Ko H.Y. (2021). Utilizing Pulmonary Function Parameters to Predict Dysphagia in Individuals With Cervical Spinal Cord Injuries. Ann. Rehabil. Med..

[3] Kirshblum S., Johnston M.V., Brown J., O’Connor K.C., Jarosz P. (1999). Predictors of dysphagia after spinal cord injury. Arch. Phys. Med. Rehabil..

[4] Abel R., Ruf S., Spahn B. (2004). Cervical spinal cord injury and deglutition disorders. Dysphagia.

[5] Chaw E., Shem K., Castillo K., Wong S.L., Chang J. (2012). Dysphagia and associated respiratory considerations in cervical spinal cord injury. Top. Spinal Cord Inj. Rehabil..

[6] Zakrasek E.C., Nielson J.L., Kosarchuk J.J., Crew J.D., Ferguson A.R., McKenna S.L. (2017). Pulmonary outcomes following specialized respiratory management for acute cervical spinal cord injury: A retrospective analysis. Spinal Cord.

[7] Hayashi T., Fujiwara Y., Sakai H., Maeda T., Ueta T., Shiba K. (2017). Risk factors for severe dysphagia in acute cervical spinal cord injury. Spinal Cord.

[8] Andrew S.A., Sidhu K.S. (2007). Airway changes after anterior cervical discectomy and fusion. J. Spinal Disord. Tech..

[9] Kepler C.K., Rihn J.A., Bennett J.D., Anderson D.G., Vaccaro A.R., Albert T.J., Hilibrand A.S. (2012). Dysphagia and soft-tissue swelling after anterior cervical surgery: A radiographic analysis. Spine J..

[10] Ihalainen T., Rinta-Kiikka I., Luoto T.M., Thesleff T., Helminen M., Korpijaakko-Huuhka A.M., Ronkainen A. (2018). Risk factors for laryngeal penetration-aspiration in patients with acute traumatic cervical spinal cord injury. Spine J. Off. J. N. Am. Spine Soc..

[11] Muss L., Wilmskoetter J., Richter K., Fix C., Stanschus S., Pitzen T., Drumm J., Molfenter S. (2017). Changes in Swallowing After Anterior Cervical Discectomy and Fusion With Instrumentation: A Presurgical Versus Postsurgical Videofluoroscopic Comparison. J. Speech Lang. Hear. Res..

[12] Ziegler J.P., Davidson K., Cooper R.L., Garand K.L., Nguyen S.A., Yuen E., Martin-Harris B., O’Rourke A.K. (2021). Characterization of dysphagia following anterior cervical spine surgery. Adv. Commun. Swallowing.

[13] Miles A., Jamieson G., Shasha L., Davis K. (2021). Characterizing dysphagia after spinal surgery. J. Spinal Cord Med..

[14] Seidl R.O., Nusser-Muller-Busch R., Kurzweil M., Niedeggen A. (2010). Dysphagia in acute tetraplegics: A retrospective study. Spinal Cord.

[15] Sura L., Madhavan A., Carnaby G., Crary M.A. (2012). Dysphagia in the elderly: Management and nutritional considerations. Clin. Interv. Aging.

[16] Yu K.J., Moon H., Park D. (2018). Different clinical predictors of aspiration pneumonia in dysphagic stroke patients related to stroke lesion: A STROBE-complaint retrospective study. Medicine.

[17] Park D., Oh Y., Ryu J.S. (2016). Findings of Abnormal Videofluoroscopic Swallowing Study Identified by High-Resolution Manometry Parameters. Arch. Phys. Med. Rehabil..

[18] Everton L.F., Benfield J.K., Michou E., Hamdy S., Bath P.M. (2022). Reliability of the Penetration-Aspiration Scale and Temporal and Clearance Measures in Poststroke Dysphagia: Videofluoroscopic Analysis from the Swallowing Treatment using Electrical Pharyngeal Stimulation Trial. J. Speech Lang. Hear. Res..

[19] Chang M.C., Choi H.Y., Park D. (2022). Usefulness of the Modified Videofluoroscopic Dysphagia Scale in Determining the Allowance of Oral Feeding in Patients with Dysphagia Due to Deconditioning or Frailty. Healthcare.

[20] Lee B.J., Eo H., Park D. (2021). Usefulness of the Modified Videofluoroscopic Dysphagia Scale in Evaluating Swallowing Function among Patients with Amyotrophic Lateral Sclerosis and Dysphagia. J. Clin. Med..

[21] Lee B.J., Eo H., Lee C., Park D. (2021). Usefulness of the Modified Videofluoroscopic Dysphagia Scale in Choosing the Feeding Method for Stroke Patients with Dysphagia. Healthcare.

[22] Chang M.C., Lee C., Park D. (2021). Validation and Inter-rater Reliability of the Modified Videofluoroscopic Dysphagia Scale (mVDS) in Dysphagic Patients with Multiple Etiologies. J. Clin. Med..

[23] Butler S.G., Markley L., Sanders B., Stuart A. (2015). Reliability of the penetration aspiration scale with flexible endoscopic evaluation of swallowing. Ann. Otol. Rhinol. Laryngol..

[24] Bluvshtein V., Front L., Itzkovich M., Aidinoff E., Gelernter I., Hart J., Biering-Soerensen F., Weeks C., Laramee M.T., Craven C. (2011). SCIM III is reliable and valid in a separate analysis for traumatic spinal cord lesions. Spinal Cord.

[25] Park H.Y., Lee H., Koh W.J., Kim S., Jeong I., Koo H.K., Kim T.H., Kim J.W., Kim W.J., Oh Y.M. (2016). Association of blood eosinophils and plasma periostin with FEV1 response after 3-month inhaled corticosteroid and long-acting beta2-agonist treatment in stable COPD patients. Int. J. Chron. Obstruct Pulmon Dis..

[26] Choi W.H., Shin M.J., Jang M.H., Lee J.S., Kim S.Y., Kim H.Y., Hong Y., Kim C., Shin Y.B. (2017). Maximal Inspiratory Pressure and Maximal Expiratory Pressure in Healthy Korean Children. Ann. Rehabil. Med..

[27] Schuld C., Franz S., Bruggemann K., Heutehaus L., Weidner N., Kirshblum S.C., Rupp R., EMSCI Study Group (2016). International standards for neurological classification of spinal cord injury: Impact of the revised worksheet (revision 02/13) on classification performance. J. Spinal Cord Med..

[28] Roberts T.T., Leonard G.R., Cepela D.J. (2017). Classifications In Brief: American Spinal Injury Association (ASIA) Impairment Scale. Clin. Orthop. Relat. Res..

[29] Kobayashi S., Miyata K., Tamura S., Takeda R., Iwamoto H. (2022). Cut-off values and sub-items of the Berg Balance Scale for walking-aid use in hospitalized older adults with a hip fracture: A retrospective analysis. Physiother. Theory Pract..

[30] Riley L.H., Vaccaro A.R., Dettori J.R., Hashimoto R. (2010). Postoperative dysphagia in anterior cervical spine surgery. Spine.

[31] Villavicencio A.T., Rajpal S., Nelson E.L., Beasley K., Kantha V., Burneikiene S. (2021). Local Retropharyngeal Space Anesthetic for Dysphagia Reduction after Anterior Cervical Discectomy and Fusion Surgery: A Single-Center, Prospective, Randomized, Double-Blinded, Placebo-Controlled Clinical Trial. World Neurosurg..

[32] Cheung J.P., Luk K.D. (2016). Complications of Anterior and Posterior Cervical Spine Surgery. Asian Spine J..

[33] Anderson K.K., Arnold P.M. (2013). Oropharyngeal Dysphagia after anterior cervical spine surgery: A review. Global Spine J..

[34] Haws B.E., Khechen B., Patel D.V., Bawa M.S., Ahn J., Bohl D.D., Mayo B.C., Massel D.H., Guntin J.A., Cardinal K.L. (2018). Impact of local steroid application in a minimally invasive transforaminal lumbar interbody fusion: Results of a prospective, randomized, single-blind trial. J. Neurosurg. Spine.

[35] Pedram M., Castagnera L., Carat X., Macouillard G., Vital J.M. (2003). Pharyngolaryngeal lesions in patients undergoing cervical spine surgery through the anterior approach: Contribution of methylprednisolone. Eur. Spine J..

[36] Song K.J., Lee S.K., Ko J.H., Yoo M.J., Kim D.Y., Lee K.B. (2014). The clinical efficacy of short-term steroid treatment in multilevel anterior cervical arthrodesis. Spine J..

[37] Jeyamohan S.B., Kenning T.J., Petronis K.A., Feustel P.J., Drazin D., DiRisio D.J. (2015). Effect of steroid use in anterior cervical discectomy and fusion: A randomized controlled trial. J. Neurosurg. Spine.

[38] Zhu B., Xu Y., Liu X., Liu Z., Dang G. (2013). Anterior approach versus posterior approach for the treatment of multilevel cervical spondylotic myelopathy: A systemic review and meta-analysis. Eur. Spine J..

[39] Gok B., Sciubba D.M., McLoughlin G.S., McGirt M., Ayhan S., Wolinsky J.P., Bydon A., Gokaslan Z.L., Witham T.F. (2008). Surgical treatment of cervical spondylotic myelopathy with anterior compression: A review of 67 cases. J. Neurosurg. Spine.

[40] Bazaz R., Lee M.J., Yoo J.U. (2002). Incidence of dysphagia after anterior cervical spine surgery: A prospective study. Spine.

